# The Imbalance Between Tumor Immunosurveillance and Tumor Immune Escape in Glioblastoma

**DOI:** 10.1111/imcb.70153

**Published:** 2026-08-19

**Authors:** Sarah J. MacDonald, Matthew Drill, Padmakrishnan C. Jayakrishnan, Richard P. Sequeira, Terence J. O'Brien, Rosalind L. Jeffree, Mastura Monif

**Affiliations:** ^1^ Department of Neuroscience, School of Translational Medicine, Faculty of Medicine, Nursing and Health Sciences Monash University Melbourne Victoria Australia; ^2^ Department of Neurology Alfred Health Melbourne Victoria Australia; ^3^ Department of Neurology Melbourne Health Parkville Victoria Australia; ^4^ Department of Neurosurgery Alfred Health Melbourne Victoria Australia; ^5^ Department of Surgery Monash University Melbourne Victoria Australia

**Keywords:** glioblastoma, immune escape, immune evasion

## Abstract

Cancer immunosurveillance is the immune‐mediated identification and elimination of malignant cells and is an essential mechanism to inhibit cancer growth. However, during cancer progression, tumors evolve various strategies to evade effective immune‐mediated eradication, a process termed tumor immune escape. Glioblastoma (GBM) is the most common form of primary brain cancer and illustrates the delicate balance between immune escape and effective immunosurveillance, and the subsequent consequences upon acquiring various strategies of evasion. GBM exhibits several strategies that drive tumor immune escape, including downregulating antigen presentation, inhibiting immune cell function, and fostering immunosuppression. This is further amplified by the tumor‐promoting effects of other tumor microenvironment constituents, such as neurons and astrocytes, and the immune system's dual role in tumor suppression and tumor persistence. Collectively, this facilitates immunologically unchecked outgrowth, manifesting as a highly aggressive, treatment‐resistant disease with a universally lethal outcome for patients with GBM.

## Graphical abstract

(Left)The identification and elimination of tumour cells is enabled by the infiltration of functional innate and adaptive immune effectors into the TME, such as natural killer cells (NK cells) and cytotoxic T cells (CD8 T cells). Effective innate and adaptive immune responses facilitate immunological control of malignant outgrowth. (Right) Tumour immune escape mechanisms in GBM include the downregulation and impairment of antigen presentation 13–16, 25, 26, the low infiltration and dysfunction of effective immune cells (CD4 and CD8 T‐cells, and NK cells 73 74, and the presence of various immunosuppressive factors in the TME 80, 81. Various components of the GBM TME further support glioma cell invasion and proliferation by releasing growth factors and angiogenic molecules, including ECM‐degrading enzymes, into the TME. This includes tumour‐associated macrophages/microglia, neurons, of which constitute a significant portion of the GBM tumour mass. Collectively, uncontrolled proliferation and invasion in GBM are enabled by various immune‐escape mechanisms, alongside the tumour‐promoting effects of TME cell populations. Created in https://BioRender.com.

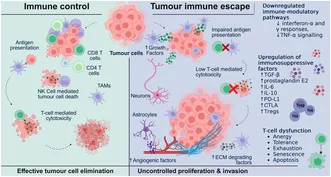

## 1 Introduction

Gliomas are a heterogeneous group of central nervous system (CNS) malignancies. Among gliomas, glioblastoma (GBM) is recognized as the most aggressive and most common primary brain cancer, accounting for up to 52.2% of all CNS malignancies [1]. GBM is characterized by rapid and extensive infiltration into the brain, and a 5‐year survival rate of only 7.0% [1]. The current standard of care for GBM comprises the Stupp protocol [2]. This involves maximally safe surgical resection and targeted cranial radiotherapy, with concurrent and adjuvant chemotherapy using temozolomide [2]. However, this protocol has not advanced since its introduction in 2005 [2]. Furthermore, as illustrated in Figure 1, there has been no significant improvement in patient outcomes despite numerous clinical trials conducted over the past 2 decades.

**FIGURE 1:**
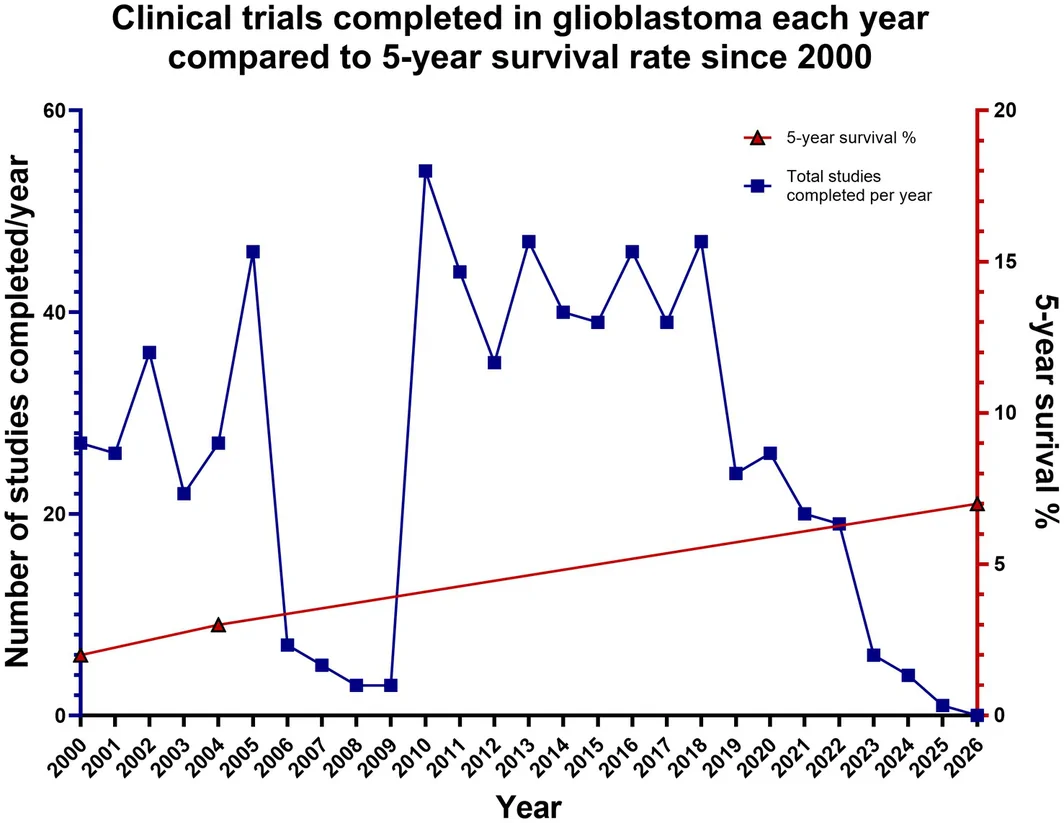
Number of clinical trials completed in glioblastoma between 2000 and 2026, compared with the 5‐year survival rate (%) for patients during this time. The 5‐year survival rate for patients with glioblastoma was ~2%–3% between 2000 and 2006 [3]. This has only marginally improved to 7% in 2025 [1], despite the numerous studies that have been completed during this time. The number of completed trials each year was sourced from the ClinicalTrials.gov registry of clinical trials, maintained by the US National Library of Medicine at the National Institutes of Health (for more information, see clinicaltrials.gov). Such numbers only represent studies within this database.

Immunosurveillance is mediated by components of both the innate and adaptive immune systems [4, 5, 6]. This concept has revolutionized cancer biology, as reflected by the advent and success of immune‐modulating therapies for various malignancies. However, due to multiple mechanisms of immune evasion, immunotherapies have thus far shown limited efficacy in GBM patients. This review aims to highlight mechanisms underlying tumor immune escape in GBM, as well as the contributions of various components of the tumor microenvironment (TME) to tumor proliferation and invasion.

## 2 Tumor Immune Escape

Intrinsic tumor‐inhibiting mechanisms identify and repair genetic alterations that may facilitate uncontrolled cell proliferation [7]. In GBM, malignant growth is preceded by a series of genetic aberrations that evade these repair mechanisms, either by activating oncogenic pathways or by inactivating key tumor suppressor genes. Such events include mutations in phosphatase and tensin homolog deleted on chromosome 10, tumor protein 53, neurofibromin 1, telomerase reverse transcriptase activation, ATP‐dependent helicase, cyclin‐dependent kinase inhibitor 2A/2B deletion, and disrupted cell cycle regulators [8]. Furthermore, several oncogenic genes/signaling pathways are dysregulated in GBM, such as receptor tyrosine kinase (RTK includes mesenchymal‐epithelial transition factor; MET, epidermal growth factor receptor; EGFR and platelet‐derived growth factor receptor alpha; PDGFRα), the RAS–ERK pathway (RAS–RAF–MEK–ERK or MAPK pathway), and phosphoinositide‐3 kinase/protein kinase B (Akt)/mammalian target of rapamycin (PI3K/AKT/mTOR) or PI3K/PTEN/AKT/mTOR pathway [8]. If intrinsic tumor‐inhibiting mechanisms are evaded, innate and adaptive immune responses are activated to identify and eliminate nascent tumor cells. Tumor‐immune interactions may exhibit a steady‐state balance, in which immune responses contain but do not completely prevent tumor cell proliferation [9]. However, if host immunity fails to eliminate malignant cells faster than they expand, this may reflect the development of mechanisms to evade effective immune responses. Thus, the failure of host immunity to control malignant outgrowth represents tumor immune escape [7, 10]. The fragile relationship between immunosurveillance and uncontrolled outgrowth during this process, and the requirement for functional host immunity, is exemplified by the markedly increased risk of tumor development in immunosuppressed transplant recipients [11, 12, 13]. In other cancers, metastasis and progression of the primary tumor site reflect tumor immune escape. In GBM, immunologically unchecked outgrowth manifests as a highly aggressive, treatment‐resistant disease that ultimately results in recurrence. Acquired evasion strategies in GBM comprise complex, dynamic processes, and interactions discussed below.

## 3 Antigen Presentation

Effective immunosurveillance requires both innate and adaptive immune responses to drive tumor‐specific adaptive immunity. However, GBM evades T cell‐mediated antitumor responses by downregulating or impairing antigen presentation in the TME [14, 15, 16, 17]. CD8 cytotoxic T cells are the primary immune effectors that recognize and destroy tumor cells that possess major histocompatibility complex (MHC) Class I antigens, while CD4 helper T cells recognize antigens presented by MHC Class II (MHCII) molecules and aid in amplifying immune responses [18]. The rejection of intracerebral tumor grafts expressing MHC Class I (MHC I) in murine models, and adherence of MHCI‐absent grafts, illustrates the importance of MHC expression during this process [19]. The downregulation of MHCI expression has been demonstrated in glioblastoma stem cells and in GBM patients [14, 15]. This has also been observed in invading glioma cells from patient‐derived tumors (MHCI), and in tumor cells derived from an invasive murine glioma model (GL261) [16]. Similarly, decreased expression of both MHC Class I and Class II has been observed in migrating human LN229 glioma cells [16]. By contrast, higher MHC expression has been associated with delayed tumor recurrence in some patients [15], suggesting a prognostic role for MHC levels in GBM. Hence, downregulation of MHC may be a key tumor immune‐escape mechanism that supports glioma invasion and recurrence.

To be recognized by cytotoxic T cells, tumor antigens must be cross‐presented by antigen‐presenting cells (APCs) via MHC molecules. Tumor‐associated microglia/macrophages (TAMs) constitute both tissue‐resident microglia and peripheral bone marrow‐derived macrophages (BMDMs) recruited to the tumor site [20, 21]. Microglia exhibit low MHCII expression at rest and function as poor antigen‐presenting cells (APCs) [22]. However, under inflammatory conditions, microglia upregulate MHCII expression to function as APCs [23], as demonstrated in human and animal models of brain tumors, suggesting their APC function in gliomas [24, 25]. However, the GBM TME has been demonstrated to downregulate microglial MHC expression and additionally impair their antigen presentation [17]. Inactivity of microglia and macrophages has been reported in the RG2 rat glioma model including low expression of costimulatory (B7.1) and MHCII molecules [17]. However, microglial cultures grown in the absence of C6 tumors exhibit increased expression of MHC and costimulatory molecules, suggesting glioma‐associated factors impede their APC function [26]. Both lymphocyte activation and MHC II expression in microglia are similarly downregulated when microglia are grown in medium derived from 9L or C6 glioma cells [26]. Hence, glioma‐associated factors may additionally impair lymphocyte function. In line with this, microglia (derived from neonatal rats) have been reported to fail to present C6 glioma cells to cytotoxic T lymphocytes and to further inhibit T‐cell‐mediated cytotoxicity of C6 glioma cells in higher density microglial cultures [27]. Therefore, microglial APC function may be impaired in the rat C6 glioma model and may also inhibit other immune effectors in the TME [27]. The exact mechanism by which GBM downregulates MHC expression in the TME remains unclear, though the presence of several immunosuppressive factors likely contributes. Recent data have illustrated the broader impact of impaired MHC‐II‐restricted antigen presentation on brain tumor immunosurveillance [28]. Specifically, MHCII expression on blood‐borne myeloid populations contributes to the persistence of cytotoxic T‐cell responses, with subsequent loss of expression driving CD8 T‐cell dysfunction via increased chromatin accessibility and expression of Tox, an important gene associated with T‐cell exhaustion [28]. Hence, MHCII expression in this immune population may be further required to maintain functional cytotoxic T‐cell phenotypes.

Tumor mutational burden refers to the number of nonsynonymous somatic mutations within a tumor sample and is often associated with genomic instability [29]. Higher mutational burdens are proposed to lead to an increased number of neoantigens within the tumor environment, thereby more effectively initiating adaptive immune responses. Tumor mutational burden has thus been utilized as a proxy for patient responses to immunotherapy across a range of cancers [30]. Various high‐mutational‐burden tumors have demonstrated significantly better clinical responses and survival following immunotherapy, including anti‐programmed death‐ligand 1 (PDL1) therapy in non‐small cell lung cancer and anti‐cytotoxic T‐lymphocyte‐associated protein 4 (CTLA‐4) therapy for melanoma [30, 31]. However, in contrast to other malignancies, associations between higher mutational burden and improved clinical outcomes have not been established in glioma patients following immunotherapy [30, 32].

The discrepancy between mutational load and immunotherapy responses in GBM may arise from several features unique to this malignancy. While GBM is characterized by significant intra‐ and intertumoral heterogeneity, these tumors paradoxically exhibit homogeneous mutational landscapes compared with other malignancies. GBM harbors a median of 2,2 somatic mutations per megabase (Mb) compared with 12.9 in melanoma and 9.9 in lung squamous cell carcinoma [33], thereby potentially yielding fewer neoantigens in the TME. While TMZ commonly drives hypermutated states in glioma [34, 35], it is well documented that this occurs without therapeutic advantage for immunotherapy [36]. Studies instead indicate that higher mutation rates are correlated with more aggressive disease states and shorter survival in patients [37]. Interesting animal data have indicated that TMZ‐induced hypermutation in glioblastoma stem cells (GL261 glioma cell line) elicits stronger immune responses than unhypermutated tumor cells when injected subcutaneously into immunocompetent mice (B57BL/6) [38]. However, when hypermutated glioblastoma stem cells were injected orthotopically into the same mice, effective immune responses were not induced [38], and additional immunotherapy (PD‐1 inhibitor) did not alter overall survival or tumor formation rates in these mice [38]. Hence, hypermutated glioblastoma stem cells could be more immunogenic, but fail to produce effective immune responses at the site of the brain. This is consistent with the discordance between mutational burden and its correlation to immunotherapy responses in GBM. However, this study used xenograft tumor formation as a proxy for immune responses, where subcutaneous rejection of xenografted tumors may equally arise from factors unrelated to host immunity, such as mouse strain or xenograft method.

Additionally, TMZ‐induced hypermutation may not necessarily equate to the expansion of immunogenic neoantigens, but may instead lead to the selection of more resistant, less immunogenic subclonal mutations across the tumor mass. This is reflected in the persistence of glioblastoma stem cells following chemotherapy and their contribution to recurrence and treatment resistance in patients, including through the activation and upregulation of DNA damage checkpoint responses and DNA repair capacity [39]. Conversely, some evidence suggests that a higher number of neoantigens may still elicit weaker immune responses than a small population of highly immunogenic entities [40]. This suggests neoantigen quality supersedes quantity in specific contexts, a finding that may also apply to GBM [41]. However, dysfunction of various innate and adaptive immune constituents within the TME likely inhibits antitumor responses, irrespective of antigen quantity or quality. In line with this, recent data have correlated immune checkpoint inhibition (ICI) responses with MHCII expression on blood‐borne myeloid cells, but not microglia, exemplifying the nuanced nature of ICI responses to specific features of GBM [38]. Overall, the mechanisms that exclude GBM from the therapeutic benefits of mutational burden likely converge with the various factors that render this disease resistant to treatment. Nonetheless, notable advancements in in silico predictions and modeling of antigen immunogenicity, alongside epitope specificity of T‐cell receptors, are likely to benefit future design of immunotherapies, such as Chimeric Antigen Therapy [42]. This may have therapeutic potential for GBM, provided other immune cell dysfunctions are addressed.

## 4 Immune Cell Dysfunction

The GBM TME features numerous infiltrating immune cells, which constitute the most significant cellular component of the TME, accounting for up to 50% of the tumor mass in some patients [43]. However, tumor immune escape is enabled by their dysfunction, in combination with the limited infiltration of effective immune cells in the GBM TME. Myeloid cells are the most abundant immune cells, predominantly comprising TAMs, while myeloid‐derived suppressor cells, dendritic cells, and neutrophils demonstrate lower infiltration [20]. By contrast, despite the infiltrative capacity of T lymphocytes into CNS tumors, GBM is characterized by a low abundance of lymphoid populations [44]. Moreover, they are frequently dysfunctional due to various GBM cell‐induced mechanisms that lead to tolerance, anergy, senescence, or exhaustion [45].

In addition to their limited infiltration, T cells within the glioma milieu exhibit classical exhaustion phenotypes and hypofunctionality [46]. The presence of immunosuppressive factors, upregulation of inhibitory molecules, and nutrient deficits within a hypoxic tumor microenvironment are significant drivers of this dysfunction [47]. Immune tolerance is an essential mechanism to prevent deleterious autoimmunity. Central tolerance mechanisms involve the elimination of developing T cells with excessive affinity for MHC complexes [48]. Conversely, self‐reactive T cells outside the thymus are controlled via peripheral deletion, T‐cell suppression, and the induction of cellular hypofunctionality [49]. Immune checkpoints are co‐stimulatory and co‐inhibitory molecules that regulate immune function and are critical molecules for this process [50]. Such checkpoints are upregulated in the glioma milieu. For instance, there are high levels of Fas ligand (FasL) on infiltrating T cells, which co‐localize with FasL‐expressing glioma cells [51], leading to an upregulation of T‐cell apoptosis. Regulatory T cells (Treg) are additional modulators of tolerance, as they actively inhibit the activation and proliferation of prospective self‐reactive T cells [52]. Similarly, there is a substantial proportion of infiltrating Treg populations within the glioma, leading to excessive T‐cell suppression and, in turn, tolerance [53]. Various soluble factors in the TME are likely to promote the proliferation of this immunosuppressive population, as demonstrated in Treg populations treated with GBM‐conditioned media [54]. For example, upregulation of the immune checkpoint molecule PD‐1 has been shown to promote Treg expansion and maintenance in peripheral leukocyte populations derived from GBM patients [55].

T‐cell anergy describes the persistent inactivity and functional unresponsiveness of T cells that is acquired following T‐cell receptor interaction in the absence of costimulatory signals [45]. Specifically, anergic T cells release insufficient interleukin (IL)‐2 to support their proliferation, a defect that may be reversed upon stimulation with exogenous IL‐2 [56]. Such a cellular state has been documented in GBM patients [57]. Specifically, infiltrating lymphocytes produce significantly less IL‐2 and interferon‐gamma than peripheral blood lymphocytes, alongside impaired IL‐2 receptor function and responsiveness despite direct stimulation [57]. Conversely, T‐cell senescence refers to a hypofunctional state of T cells characterized by low proliferative activity [58]. This is thought to be driven by telomere erosion, excessive proliferative activity, or increased reactive oxygen species in an inflammatory TME, such as GBM [45]. Senescent T cells also accumulate with normal aging [59], although few studies have evaluated their presence in GBM. However, one study has described a large population of senescent T cells in blood and tumor samples from GBM patients [60]. These were identified by loss of CD28 expression on CD8 T cells, an essential co‐stimulatory molecule required for CD8 T‐cell activation [61]. Interestingly, all peripheral T‐cell populations expressed a single checkpoint, PD‐1, with significantly higher expression in CD28‐negative populations [60]. However, tumor‐infiltrating senescent populations did not exhibit single‐checkpoint expression in tumor tissue, indicating distinct peripheral populations [60]. Given GBM has a higher prevalence in older individuals, the authors conclude that this malignancy likely amplifies the age‐related accumulation of senescent T cells [60]. However, due to the paucity of studies that evaluate T‐cell senescence, further validation and exploration are required. This may have significant therapeutic value, as it may offer mechanistic insight into why current immunotherapy approaches fail. Sufficient functional T cells in the TME are required to rescue antitumor responses through immunotherapy.

Exhausted T cells describe a subset of lymphocytes that feature progressive loss of effector functions and upregulation of inhibitory cell‐surface molecules [62]. This exhaustion phenotype is prevalent in the glioma milieu [63]. It has been proposed that tolerized T‐cell states may arise from improper cross‐priming by APCs [64]. Growing evidence suggests that T cells' localization to peripheral and CNS tumors is dictated by APCs at the site of antigen capture rather than in lymphoid organs [65, 66]. This further underscores the significance of functional antigen presentation for T‐cell function. Dendritic cells (DCs) are innate immune cells that coordinate immune responses by expressing co‐stimulatory molecules and releasing various cytokines within lymphoid organs [67]. In the CNS, DCs can serve critical functions in the capture and presentation of antigens and are therefore an important immune population for priming naïve T cells [68]. However, GBM patients have fewer DCs in tumor tissues and in peripheral blood, and those that are present display less functional phenotypes [69]. Such conditions are conducive to the conditions of T‐cell exhaustion. Another study has demonstrated that IL‐10 signaling from defined myeloid populations contributes to T‐cell exhaustion, which can be rescued by inhibiting JAK–STAT in vivo and in vitro (mediated by reduced release of this cytokine) [70]. This suggests that targeting this signaling pathway may alleviate T‐cell exhaustion, albeit more data are needed.

Recent multi‐omics analyses have highlighted distinct transcriptomic signatures regarding exhausted or tolerized CD8 T cells that vary across tumor types, specifically depending on their inherent antigenicity [63]. Compared with immunologically hot tumors, tolerized T cells within the GBM tumor microenvironment have been shown to exhibit low cell cycle activity and an increased tendency toward cell death [63]. Furthermore, this T‐cell compartment exhibits decreased effector and co‐stimulatory signaling, alongside increased enrichment of self‐recognizing T‐cell receptor epitopes [63]. Such data summarize and reiterate hypofunctional states distinct to the GBM lymphoid populations.

Despite impaired T‐cell responses (T‐cell dysfunction), additional antitumor immune responses and effectors may be present in the TME. However, several of these have additionally been shown to be impaired in GBM. Natural killer (NK) cells are innate lymphoid cells that mediate cytotoxicity against cancer cells and drive adaptive immune responses by releasing cytokines and chemokines [71]. While low MHCI expression can activate NK cells [72], NK cell representation in the GBM TME is notoriously low. An examination of GBM tissue biopsies has reported that NK cells comprise only 2.11% of all tumor‐infiltrating immune cells [73]. Furthermore, the consequences of low NK cell infiltration are likely compounded by various glioma‐associated factors that suppress or impair NK cell function. Glioma has been associated with downregulation of natural killer Group 2, member D (NKG2D), a key receptor that regulates the cytotoxic functions of NK and CD8 T cells [71]. Transforming growth factor‐beta (TGF‐β) derived from the serum of glioma patients has been reported to convert NKG2D cells from an active to an inactive state [74]. Additional impairment of antitumor responses is further supported by recent data showing that glioma cell activation (via phorbol myristate acetate) can induce immune‐tolerant natural killer T cells (NKT cells) [75], a subcategory of T cells that shares characteristics of both T and NK cells [76]. Glioma cell miR‐92A, a family of microRNAs considered to participate in tumorigenesis, derived from patient cultures or U87 glioma cells, was demonstrated to differentiate a fraction of NKT cells that suppressed CD8 T‐cell proliferation [75]. Collectively, these observations highlight the widespread and intertwined dysfunction of both innate and adaptive immune cells in the GBM TME. Interestingly, the frequency of NK and CD8 T cells expressing NKG2D in GBM patients has been shown to increase significantly (from 43.0% to 70%) following surgical resection and correlate with glioma burden [74, 75], highlighting the potential clinical implications of glioma‐mediated NK cell dysfunction in GBM.

## 5 Immunosuppression

### 5.1 Local Immunosuppression

Immunosuppression facilitates tumor immune escape by inhibiting antitumor responses, thereby allowing unchecked tumor cell survival, proliferation, and invasion. In the local TME, GBM can suppress the immune response by upregulating various immunosuppressive factors, including TGF‐β [77], prostaglandin E2 [78], IL‐6 [78], and IL‐1 [79]. Glioma‐derived TGF‐β can exert immunosuppressive effects on infiltrating lymphocytes by inhibiting T‐cell proliferation [80], whereas glioma‐derived IL‐10 has been demonstrated to dampen antitumor responses by reducing interferon‐gamma and tumor necrosis factor‐alpha production [81]. Additionally, glioma‐derived factors have been associated with the upregulation of the expansion and function of immunosuppressive effector cells in the TME. Soluble factors secreted by GBM cell lines have been demonstrated to facilitate recruitment, survival, and expansion of Tregs [54], whereas the release of placental growth factor containing vesicles from glioma cells has been linked with upregulating the function of immunosuppressive regulatory B cells [82].

As mentioned, immune checkpoints regulate immune activation and inhibition [50]. Accordingly, the upregulation of these molecules offers an effective mechanism of tumor immune escape. A key immune checkpoint molecule that facilitates GBM immune evasion is PD‐L1, a transmembrane glycoprotein of the B7 family of co‐stimulatory molecules [83]. Activation of PD‐L1 can promote immunosuppression by inhibiting CD8 T‐cell proliferation and function, and by upregulating function of Tregs [83]. In GBM, PD‐L1 expression is upregulated on several immune cell types. Upregulated PD‐L1 expression has been demonstrated in both peripheral CD4 and CD8 T cells of glioma patients and was associated with disease progression [84]. Hence, immune cell expression of PD‐L1 may be an important factor in glioma progression. Elevated PD‐L1 has also been reported in circulating monocytes and TAMs in glioma patients [83], as well as normal monocytes exposed to glioma‐conditioned media [83]. Furthermore, co‐culture experiments have demonstrated that monocytes/TAMs expressing PD‐L1 induce apoptosis in peripheral T cells isolated from the same patients [83]. Of note, the magnitude of this effect increased with higher levels of PD‐L1 expression in monocytes/TAMs, further underscoring the potential impact of PD‐L1 on disease progression. Elevated PD‐L1 levels in the GBM TME are likely driven by several mechanisms. Upregulated expression has been linked with autocrine/paracrine IL‐10 signaling in circulating monocytes/TAMs [83]. Alternatively, loss of PTEN and activation of the PI3K pathway have been linked to increased PD‐L1 expression in GBM specimens [85].

### 5.2 Systemic Immunosuppression

In addition to a highly immunosuppressive TME, GBM patients exhibit profound systemic immunosuppression. Various studies have highlighted reduced T lymphocyte counts and function in GBM patients, most notably of CD4 populations [86, 87]. CD4 T cell number deficiency has been described to reach numbers as low as those seen in patients with acquired immunodeficiency syndrome [88]. T cells have instead been described to sequester in bone marrow [88, 89], with a significantly higher proportion of immunosuppressive CD4 Tregs [90]. Involution of lymphoid organs with T‐cell deficiency has also been demonstrated in GBM patients [88] and replicated in the GL261 [89, 91] and SMA‐560 glioma‐bearing (VM/Dk) murine glioma model [88].

How local immunosuppression may translate into systemic immunosuppression, and the mechanisms driving this phenomenon, remain poorly understood. Circulating levels of immunosuppressive cytokines in patients have been proposed to be too low to account for such systemic immunosuppression [92]. Furthermore, while conventional treatments likely exacerbate T‐cell deficiencies [93, 94], reduced counts likely arise independent of treatment [88, 90]. T‐cell lymphopenia has been reported in patients before receiving steroids or chemotherapy [90], and T‐cell deficiencies have been demonstrated in GBM patients and several mouse models of glioma that have not undergone conventional therapy [88]. Recent data from R53 tumor‐bearing mice have indicated that GBM may influence systemic myeloid differentiation in the bone marrow during tumor progression, specifically by promoting the production of neutrophils with pro‐tumor phenotypes [95]. However, whether such polarizations persist upon localization with the tumor remains unknown.

Conversely, substantial research has focused on developing accurate biomarkers in liquid biopsies for GBM [96, 97, 98]. The breadth of literature in this field is a testament to the many glioma‐associated factors that reach the periphery in a detectable manner. It is therefore highly plausible that various immunosuppressive factors or cells derived from the TME extend their immunosuppressive effects into circulation. Studies have demonstrated detection of circulating tumor cells (CTC's) in the peripheral blood of GBM patients (29/141 patients) by density‐gradient centrifugation, and the identification of EGFR amplification and gains/losses of chromosomes 7 and 10 in glial fibrillary acid protein (GFAP)‐positive cells [99]. However, while this study demonstrates that these neoplastic cells are detectable in GBM patients' blood, no significant associations were observed between their counts and clinical outcomes (such as recurrence or survival) [99]. Moreover, the capacity of tumor cells to circulate from the brain into the peripheral circulation is likely impaired in CNS malignancies, as CTCs have been reported to occur less frequently in patients with isolated brain metastases than in those with non‐isolated CNS metastases [100]. Hence, CTCs reaching the circulation may equally be of no consequence for disease trajectories, or specifically for systemic immunosuppression. Nonetheless, numerous studies support their detection from liquid biopsies [99, 101]. Alternatively, extracellular vesicles are a heterogeneous category of nanosized membrane‐bound particles, including several subgroups such as apoptotic bodies, large oncosomes, microvesicles, and exosomes [102]. Extracellular vesicles contain substantial amounts of tumor‐related proteins, lipids, and nucleic acids, including growth factors, cytokines, and membrane receptors [103]. Moreover, their surrounding double‐layer lipid membrane protects cargo content from degradation [103]. This may facilitate the systemic transfer of immunosuppressive factors from the tumor microenvironment. For instance, patient‐derived extracellular vesicles have been demonstrated to express a unique protein that inhibits antigen recognition and presentation in DCs, thereby indirectly suppressing cytotoxic T‐cell responses [104].

### 5.3 Translational Perspective

Emerging immunotherapies currently being evaluated in preclinical and clinical models for GBM include cytokine modulation [105], TAM reprogramming [106], oncolytic viruses [107], and adoptive T‐cell therapies [108]. In theory, several of these approaches are directly aimed at mitigating the various aspects of immune cell dysfunction and immunosuppression previously discussed. Examples and further details of such therapies are illustrated in Table 1. For simplicity, the prospect of cancer vaccines will be discussed in further detail to illustrate how such dysfunction may be harnessed through immunotherapy. In principle, cancer vaccines address downregulated antigen presentation and impaired T‐cell priming and function in the glioma milieu. Specifically, this therapy aims to harness specific host immunity by upregulating the presentation of tumor‐associated or tumor‐specific antigens to immune cells [105]. This is thought to enhance immunological memory and recognition of tumor antigens, thereby allowing the immune system to rapidly identify and eradicate tumor cells. Such effects may be achieved via differential vaccine designs. Peptide vaccines introduce known peptides, or antigenic proteins, to promote antigen presentation and subsequent eradication of cells bearing these molecular signatures, such as EGFR Variant III (EGFRvIII) in GBM [118]. Autologous vaccines are created by retrieving peripheral blood cells from the patient and reintroducing them after priming, either by exposing them to known antigens or by altering tumor cells with viruses [119]. Similarly, the engineering of DC vaccines involves isolating these innate immune cells from patient blood and exposing them to patient‐derived glioma antigens to activate naïve lymphocytes upon reintroduction [120]. Lastly, heat shock protein (HSP)‐peptide‐based vaccines purify HSPs from patient‐derived tumors to facilitate their interaction with APCs upon reintroduction to the host, thereby priming lymphocytes with an array of patient‐derived antigenic peptides [119].

**TABLE 1:** Current immunotherapies that are being explored in GBM.

Immunotherapy type	Description	Therapeutic potential	Example trials in GBM
Adoptive T‐cell therapies	Extract patients' own T cells, alter them to amplify immune responses, and then reinfuse them back into the patient		
Tumor‐infiltrating T‐cell therapy	Reinvigorating T cells with cytokines, or engineering them to secrete immune‐modulatory molecules	Upregulate antitumor responses or prevent T‐cell exhaustion	Exposing autologous T cells to IL‐2 before reinfusion [109] Autologous T cells engineered to secrete PD‐1 [110]
Chimeric antigen receptor‐T‐cell therapy	Genetically engineered to express receptors on their surface to target specific tumor antigens	Upregulate cytotoxic T‐cell responses specific to the tumor	IL‐13Rα2 targeted CAR‐T cells [42] EGFRvIII and EGFR‐targeted CAR‐T cells [108]
Cancer vaccines	Vaccines engineered to introduce tumor‐associated or tumor specific antigens to train immune system to recognize cancer		
Dendritic cell vaccines	Isolated DC from patient blood, and exposing them to glioma‐associated antigens before reinfusion	Upregulation antigen presentation/prime naïve lymphocytes for immunological memory	DCVax‐L‐an autologous tumor lysate DC vaccine [111]
Peptide vaccine	Synthetic short amino acid chains that resemble tumor antigens	Increase presentation of tumor proteins via MHC to cytotoxic T cells	SurVaxM—a survivin‐targeting vaccine [112]
Oncolytic viruses	Genetically modified viruses that infect and lyse tumor cells	Release of tumor‐associated antigens, damage‐associated molecular patterns, and pathogen‐associated molecular patterns to stimulate immune responses, such as the migration of APCs to cytotoxic T cells	PVSRIPO‐poliovirus/rhinovirus chimeric virus that targets glioma cells through CD155 [113]
Alternative sites of immunosuppression			
Cytokine modulation	Modulating specific cytokine levels, mainly potent antitumor molecules	Boost antitumor responses in a controlled manner	Herpes simplex virus Type 1 (HSV‐1) virus engineered to promote IL‐2 release from tumor cells [105]
Myeloid‐targeting therapy	Modulating tumor‐infiltrating myeloid populations, such as tumor associated macrophages/microglia (TAMs)	Inhibition of TAM recruitment, enhancement of their tumor phagocytic activity, and modulation of their polarization. Collectively, reducing their immunosuppressive/tumor‐supportive effects in the TME (preclinical models) [114]	Reprogramming TAMs from immunosuppressive to pro‐inflammatory phenotypes using Colony stimulating factor 1 receptor inhibition [115]
Immune checkpoint inhibition	Blocking immunosuppressive immune checkpoint molecules with monoclonal antibodies	Combats T‐cell exhaustion and hypo functionality to boost their antitumor responses	Anti‐CTLA antibody [116]. Anti‐PD‐1 antibody [117]

SurVaxM is a peptide vaccine engineered against the survivin, a critical cell‐survival protein that is highly expressed in GBM [112]. In a Phase IIa multicenter single‐arm trial, GBM patients who received adjuvant SurVaxM (in combination with granulocyte–macrophage colony‐stimulating) demonstrated a median overall survival of 25.9 months following the first dose (post‐surgery and radiotherapy with concurrent TMZ) [112]. Furthermore, SurVaxM treatment was shown to produce tumor‐specific CD8 T cells [112]. While this is a substantial increase in median overall survival compared to conventional therapy, additional treatment arms are needed to evaluate the therapeutic benefit of this vaccine. This indicates that vaccines derived from autologous or predefined antigenic entities may yield clinically meaningful improvements in patient outcomes. However, in theory, autologous antigens are likely to offer the greatest benefit, given the substantial intra‐tumoral heterogeneity observed in GBM patients.

In 2023, results from a Phase 3 clinical trial were published on the use of an autologous tumor lysate dendritic cell vaccine (DCVax‐L) in combination with conventional therapy in 331 patients with newly diagnosed or recurrent GBM [111]. Newly diagnosed patients who received DCVax‐L had a median overall survival of 19.3 months, while those receiving placebo treatment had a median overall survival of 16.5 months. For recurrent patients, median overall survival from relapse was 13.2 months, compared with 7.8 months in patients who did not receive this treatment [111]. The authors conclude that this vaccine can offer a clinically meaningful and statistically significant increase in median overall survival for both newly diagnosed and recurrent GBM. Furthermore, given its safety and tolerability in this trial, this vaccine may be suitable for use in combination with conventional treatments or, conversely, with additional immunotherapies such as ICI.

Considering the intricate dysfunction across multiple layers of immunity in GBM, a combinatorial approach to immunotherapy may supersede monotherapy. Functional immune mechanisms that cascade across both the innate and adaptive arms of host immunity are paramount to the immunological rejection of tumor. However, these are entangled rather than discrete mechanisms. Thus, merely reinvigorating a single component that depends upon a multiplicity of others may be insufficient to bolster antitumor immunity. Indeed, in addition to inflammation and toxicity risks, this approach adds further complexity to an already convoluted and treatment‐resistant landscape. Nevertheless, some clinical trials are evaluating this, including combined IL‐12 gene therapy (aimed at stimulating adaptive immune responses) and ICI (nivolumab) in recurrent GBM [121], or neoadjuvant triple immune checkpoint blockade [122] (An Australian‐led international clinical trial soon to be commenced).

## 6 Tumor Microenvironment

If antitumor immune responses are sufficient to eradicate tumor cells faster than their rate of expansion, successful immune regulation prevents tumor immune escape. However, various components of the GBM TME support tumor progression and, by extension, tumor immune escape.

### 6.1 Tumor‐Associated Microglia/Macrophages

TAMs comprise up to 50% of the tumor mass, and have historically been classified as either “classically activated” or “M1” and “alternatively activated” or “M2” phenotypes [123]. However, TAMs can exhibit a spectrum of phenotypes beyond the “M1/M2” dichotomy [124, 125, 126] and are likely to exist across a spectrum in the TME. Naïve macrophages (M0 macrophages) polarize to “M1‐like” pro‐inflammatory phenotypes in response to damage‐associated molecular patterns and pathogen‐associated molecular patterns, including toll‐like receptor 4 ligands, INF‐γ, lipopolysaccharide, or GM‐CSF factor [127]. “M1‐like” macrophages are associated with various pro‐inflammatory and tumor‐suppressing functions, such as upregulating antigen presentation and the production of proinflammatory cytokines [128], including C‐C motif chemokine ligand 2, TNF‐α, and IL‐1 beta [127, 129, 130]. By contrast, “M2‐like” anti‐inflammatory or regulatory TAMs are polarized upon exposure to anti‐inflammatory cytokines, including TGF‐β, IL‐4, IL‐10, or IL‐13, and exhibit immune‐suppressing function in the TME [128].

TAMs exhibit various tumor‐promoting functions in the GBM TME. This has been exemplified by the observation that microglial depletion markedly reduces glioma cell proliferation, invasion, or expansion of glioma cells in both in vitro [131, 132] and in vivo models [133]. TAMs release a multiplicity of cytokines and growth factors that have been shown to support glioma cell proliferation and invasion, including IL‐6, IL‐1β, stress‐inducible protein 1, and basic fibroblast growth factor [134, 135]. Among many, TGF‐β and epidermal growth factor (EGF) have been highlighted as key tumor‐promoting factors. TAM‐released EGF activates EGF receptors on glioma cells, triggering various signaling cascades that support glioma cell proliferation and survival, such as P13K/AKT/mTOR [136], a key pathway associated with various human malignancies. Conversely, selective inhibition of TGF‐β using short hairpin RNA has been demonstrated to effectively reduce glioma cell invasion in human T98G and rat C6 glioma cells [137]. The release of this growth factor has also been shown to enhance stemness in U251 glioma cells [138] and may therefore contribute to therapeutic resistance. Specifically, TAMs may support glioma cell invasion by releasing or stimulating extracellular matrix (ECM)‐degrading factors. Matrix metalloproteinase‐2 (MMP‐2) is a critical protease involved in glioma cell invasion, as this protease can break down the ECM and further activate other metalloproteases. TAMs activate and upregulate this protease by converting glioma‐derived pro‐MMP2 into MMP‐2 [131], and further upregulate MMP‐2 in glioma cells through TAM‐derived CCL5, while expressing MMP‐2 themselves [131]. Additionally, TAMs are a potent source of angiogenic factors, such as vascular endothelial growth factor A, stimulating ECM degradation and the formation of new blood vessels to facilitate angiogenesis [139]. Recently, TAMs have been shown to indirectly support vascular mimicry in GBM, a process in which tumors create their own blood vessels to support tumor progression [140, 141]. This can occur through the release of IL‐6 from TAMs, which activates the Janus kinase/signal transducer and activator of transcription signaling pathway, a key pathway that leads to pseudo‐vascularization [142].

### 6.2 Astrocytes

In addition to the main immune infiltrates, various stromal constituents of the GBM TME also facilitate tumor growth. The primary response to any brain lesion is mediated by astrocytes. In vitro data indicate that astrocytes near GBM lesions undergo reactive astrogliosis [143]. Reactive astrocytes can additionally release several growth factors and cytokines that promote GBM proliferation, migration, and infiltration. Activated astrocytes secrete high levels of IL‐6, which increases the expression of proteases that degrade the ECM, such as MMPs, thereby facilitating tumor invasion and infiltration. They can also secrete connective tissue growth factor, which can bind to integrin β1, triggering nuclear factor kappa‐light‐chain‐enhancer of activated B cells (NF‐κB) signaling and Zinc Finger E‐Box Binding Homeobox 1 to support GBM cell infiltration [144]. Connective tissue growth factor inhibition has been reported to reduce GBM cell proliferation, migration, and invasion [144, 145]. Reactive astrocytes have also been linked to a poorer prognosis [146], highlighting the contribution of astrocyte‐released factors toward glioma progression. Astrocytes also regulate cerebral blood flow and the blood–brain barrier via their end‐foot processes, which envelope cerebral blood vessels [147]. In the GBM TME, disruption of this association is an important mechanism supporting glioma cell invasion [148]. Furthermore, several astrocyte‐released and astrocyte‐expressed factors regulate blood vessel formation and stabilization to support angiogenesis, including vascular endothelial growth factor A, integrins, and ECM proteins [149].

### 6.3 Neurons

Bidirectional interactions between glioma cells and their surrounding neural architecture have also been implicated in supporting GBM progression [150]. Optogenetic stimulation of neurons within a patient‐derived xenograft murine model has been demonstrated to significantly increase GBM cell proliferation in vivo [150]. This observation has strongly implicated active neurons in gliomagenesis. The postsynaptic adhesion molecule Neuroligin 3 has since been identified as an activity‐regulated mitogen in the TME dictating glioma growth. Its presence has been reported to enhance proliferation in patient‐derived high‐grade glioma cell cultures [150], while its absence in a patient‐derived xenograft murine model has been shown to inhibit tumor formation [151]. Neuroligin 3 has been reported to activate several oncogenic pathways, including the focal adhesion kinase upstream of the PI3K‐mTOR pathway [151], which may explain its growth‐promoting effects. Similarly, brain‐derived neurotrophic factor (BDNF), a well‐characterized growth factor released in response to depolarization [152] has been identified as a potential tumor‐promoting mitogen in the GBM TME [150]. Genetic knockdown of activity‐regulated BDNF has been demonstrated to inhibit tumor growth and prolong survival in patient‐derived xenograft mouse models of pediatric GBM [153]. BDNF has additionally been demonstrated to activate various signaling pathways in glioma cells. The MAPK/ERK/MEK signaling cascade is an important pathway for several cellular processes, including proliferation, differentiation, and survival [154], and has been implicated in a significant fraction of human cancers [155]. BDNF exposure in glioma cells has been shown to activate this pathway, along with calcium/calmodulin‐dependent protein kinase II signaling, an important component of α‐amino‐3‐hydroxy‐5‐methyl‐4‐isoxazolepropionic acid (AMPAR) trafficking in neurons [156]. However, because BDNF is secreted by neuroglia and neurons, it is unclear whether its source within the TME is significant for glioma progression. Glioma cells have also been shown to upregulate genes associated with synapse function after Neuroligin 3 exposure [150]. Interestingly, gene ontology analysis of recurrent GBMs has demonstrated significant enrichment for terms related to neuronal signaling, including neurotransmitter support and chemical synaptic transmission [157]. Collectively, these observations suggest that neuronal signaling contributes to glioma growth and recurrence, mediated by the activity‐regulated release of mitogens in the glioma milieu.

Recent literature suggests that glioma cells can form functional synapses with neurons, which contributes to glioma growth. Electron microscopy data within primary GBM tissue and patient‐derived xenograft mouse models have offered structural evidence of neuron–glioma synapses [158]. Whole‐cell patch‐clamp recordings have since demonstrated that glioma cells can induce excitatory postsynaptic currents mediated by AMPAR receptors [158]. Notably, this can depolarize glioma cells, thereby promoting their proliferation, an effect mediated via AMPAR receptor activation [158]. Collectively, this indicates that gliomas exhibit canonical features of electrically functional synapses with surrounding neurons, which contribute to glioma growth. Interestingly, BDNF exposure had also been shown to increase surface levels of AMPAR subunits on glioma cells [153], linking paracrine signaling and synaptic mechanisms of tumor‐promoting effects. In further elaboration, data from intracranial recordings and tumor tissue biopsies have indicated that the degree of functional connectivity between glioma cells and their surrounding neural networks also negatively affects patient survival [159]. Glioma cells from highly connected tumors have also been shown to release substantial amounts of the synaptogenic factor thrombospondin‐1 (TSP‐1). Pharmacological inhibition of this molecule with gabapentin can reduce glioma cell proliferation and network synchrony in mice bearing high functional connectivity patient‐derived tumors [159]. Such data elaborate on the role of neuronal signaling in glioma growth and directly implicates TSP‐1 as a critical synaptic molecule mediating high‐functional neuronal connectivity [159].

Neuronal activity may promote glioma proliferation not only in an activity‐dependent or paracrine‐mediated manner but also through immune‐inhibiting mechanisms. High functional connectivity has also been shown to induce regional immunosuppression in a spatially dependent manner [160]. Recent scRNA‐seq data indicate that regions of high functional connectivity within patient samples (predetermined from MEG and MRI) show significantly lower expression of various immune‐related genes compared with regions of lower functional activity across glioma, myeloid, and lymphoid populations [160]. Further, pathway analysis and pseudo‐bulk analysis revealed downregulation of key immune‐modulatory pathways, including the inflammatory response, interferon‐α and γ responses, and TNF‐α signaling via the NF‐κB pathway, across all cell populations [160]. Intriguingly, high‐functional‐connectivity regions showed a higher proportion of anti‐inflammatory myeloid phenotypes, suggesting such conditions incite immunosuppressive myeloid phenotypes [160]. Notably, subsequent experiments utilizing the high TSP‐1‐expressing glioma cell line SB28 (as a model of high functional connectivity) indicated that knockout (KO) of this protein may alleviate immunosuppression in vivo [160]. CRISPR‐Cas9‐mediated KO of TSP‐1 in an orthotopic mouse model led to increased expression of various immune‐regulatory pathways, including antigen presentation, while also promoting infiltration by pro‐inflammatory myeloid and lymphoid populations [160]. Interestingly, inhibition of excitatory glutamatergic signaling in an SB28 orthotopic mouse model (TSP‐1 WT) utilizing antiepileptic medication prolonged survival and increased the therapeutic efficacy of ICI treatments. This indicates that targeting high functional activity, namely glutamatergic signaling, not only inhibits glioma proliferation but may also mitigate a highly immunosuppressive TME, offering compelling evidence for the use of antiepileptic drugs as an adjunct treatment in GBM patients.

Gabapentinoids are commonly prescribed for seizures and neuropathic pain [161]. Considering GBM patients often suffer from seizures, and these agents likely do not impede conventional therapy, neoadjuvant treatment with such compounds may alleviate the tumor‐promoting effects of neuronal activity. However, this concomitantly presents surgical challenges. Eloquent, highly functional regions may represent highly aggressive and/or immunosuppressive tumor regions, yet preserving such regions is imperative to prevent neurological deficits and adverse effects on patient quality of life. Nonetheless, one retrospective, multi‐institutional cohort study comprising 1072 patients has demonstrated a statistically significant improvement in overall survival among newly diagnosed patients treated with gabapentinoids following resection (16 months) compared with patients who did not receive this compound (12 months) [95]. Hence, perhaps pharmacological treatment of high functional connectivity is sufficient to confer a survival advantage. Collectively, these studies underscore the diversity of constituents that can modulate host immunity and warrant further exploration to identify additional GBM‐specific modulators for prospective therapeutic applications (Figure 2).

**FIGURE 2:**
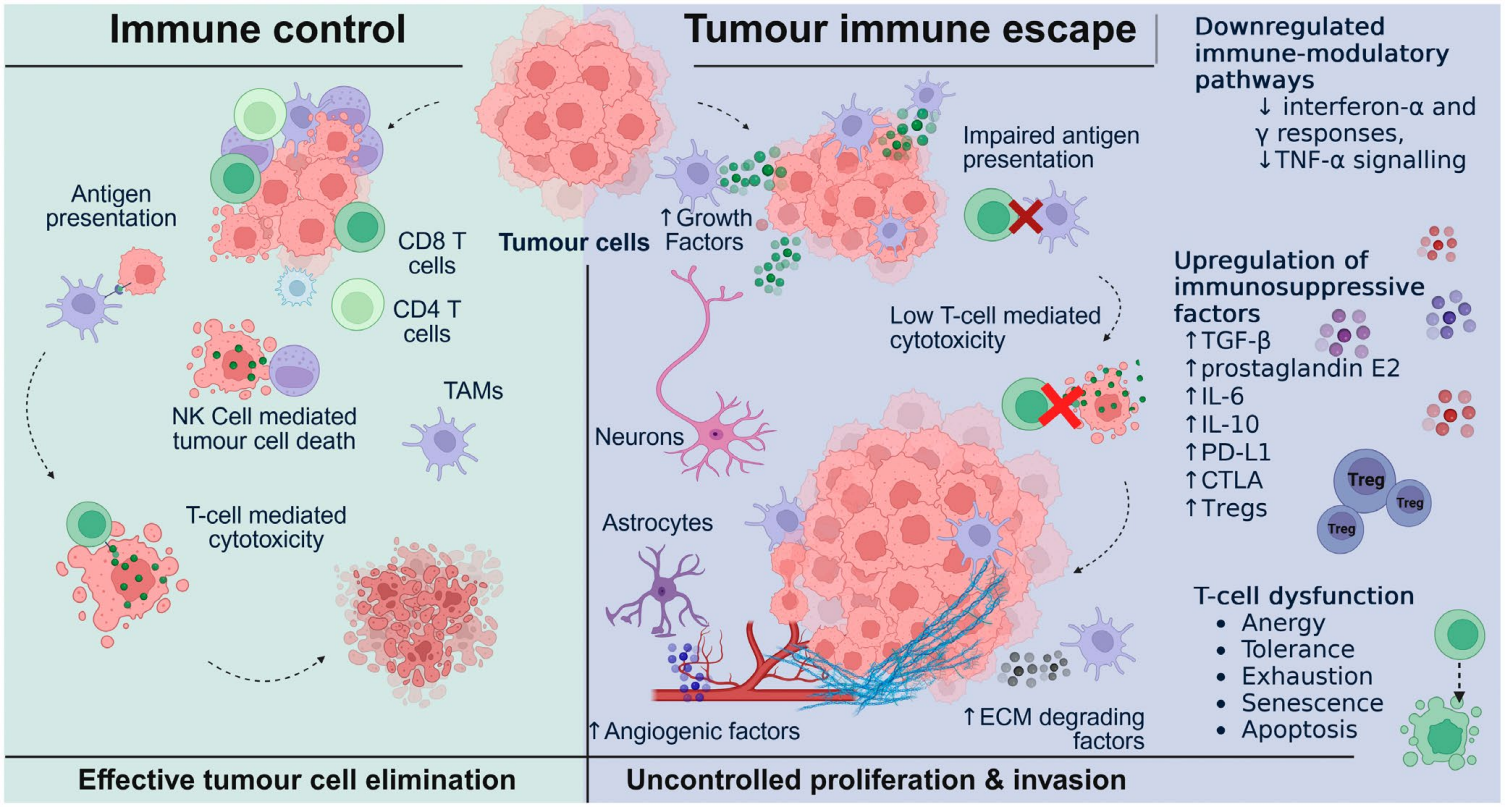
(Left) The identification and elimination of tumor cells is enabled by the infiltration of functional innate and adaptive immune effectors into the TME, such as natural killer cells (NK cells) and cytotoxic T cells (CD8 T cells). Effective innate and adaptive immune responses facilitate immunological control of malignant outgrowth. (Right) Tumor immune escape mechanisms in GBM include the downregulation and impairment of antigen presentation [14, 15, 16, 17, 26, 27], the low infiltration and dysfunction of effective immune cells (CD4 and CD8 T cells, and NK cells) [73, 74], and the presence of various immunosuppressive factors in the TME [80, 81]. Various components of the GBM TME further support glioma cell invasion and proliferation by releasing growth factors and angiogenic molecules, including ECM‐degrading enzymes, into the TME. This includes tumor‐associated macrophages/microglia and neurons, which constitute a significant portion of the GBM tumor mass. Collectively, uncontrolled proliferation and invasion in GBM are enabled by various immune‐escape mechanisms, alongside the tumor‐promoting effects of TME cell populations. Created in https://BioRender.com.

## 7 Conclusion

GBM is the most aggressive and common form of primary brain cancer. The current standard of care comprises the Stupp protocol. However, no significant improvements in patient outcomes have been observed since this protocol was implemented in 2005. The concept of immunosurveillance describes the host‐mediated eradication of tumor cells and has significantly advanced modern cancer treatment. However, such immune‐modulating therapies have shown limited success in GBM, owing to various factors within the TME that evade effective immune responses, known as tumor immune escape.

Tumor immune escape in GBM is driven by various complex, dynamic mechanisms within the glioma milieu. Effective T‐cell‐mediated responses are impaired by the downregulation or dysfunction of antigen presentation on both glioma cells and APCs within the TME. Furthermore, while the GBM tumor mass features numerous infiltrating immune cells, immune escape is enabled by the limited numbers of effective immune cells, as demonstrated by the composition of NK cells and lymphoid populations within the GBM TME. Glioma cells further suppress effective immune responses by releasing and expressing immunosuppressive factors in the TME. Such factors include various cytokines, growth factors and immune checkpoint molecules, which collectively foster a highly immunosuppressive TME. Intriguingly, GBM patients often exhibit profound systemic immunosuppression, characterized by reduced lymphocyte count and function, and involution of lymphoid organs. However, the translation of local immunosuppression to systemic immunosuppression in patients is not well understood, as such effects can occur independent of conventional therapies. Lastly, uncontrollable tumor growth is enabled by various stromal and immune constituents of the GBM TME, including TAMs, astrocytes, and neurons. These constituents facilitate glioma cell proliferation and invasion and further promote angiogenesis by releasing various tumor‐promoting factors in the TME. Collectively, this warrants research aimed at restoring immune function, while reversing the pro‐tumor effects of TME constituents, to improve and augment current treatments for patients with GBM.

## Author Contributions

**Matthew Drill:** supervision, conceptualization, writing – review and editing. **Richard P. Sequeira:** writing – review and editing. **Rosalind L. Jeffree:** writing – review and editing, supervision. **Padmakrishnan C. Jayakrishnan:** writing – review and editing. **Sarah J. MacDonald:** writing – original draft, conceptualization. **Mastura Monif:** funding acquisition, writing – review and editing, supervision, conceptualization. **Terence J. O'Brien:** writing – review and editing, supervision.

## Funding

This work was supported by 2011590.

## Conflicts of Interest

The authors declare no conflicts of interest.

## Data Availability

Data sharing not applicable to this article as no datasets were generated or analysed during the current study.
